# Interrelationship between Altered Left Ventricular Ejection Fraction and Nutritional Status in the Post-Acute Myocardial Infarction Patient

**DOI:** 10.3390/nu16132142

**Published:** 2024-07-04

**Authors:** Maria Gențiana Czinege, Victoria Nyulas, Vasile Bogdan Halațiu, Constantin Țolescu, Liliana-Oana Cojocariu, Teodora Popa, Tiberiu Nyulas, Theodora Benedek

**Affiliations:** 1 Doctoral School of Medicine and Pharmacy, “George Emil Palade” University of Medicine, Pharmacy, Science and Technology of Târgu Mureș, 540139 Târgu Mureș, Romania; maria.czinege@umfst.ro; 2 Department of Informatics and Medical Biostatistics, “George Emil Palade” University of Medicine, Pharmacy, Science and Technology of Târgu Mureș, 540139 Târgu Mureș, Romania; 3 Clinic of Cardiology, County Emergency Clinical Hospital, 540136 Târgu Mureș, Romania; bogdan.halatiu@umfst.ro (V.B.H.); cristi.tolescu95@gmail.com (C.Ț.); cjrliliana@yahoo.com (L.-O.C.); popateodoramaria@gmail.com (T.P.); theodora.benedek@umfst.ro (T.B.); 4 Department of Physiology, “George Emil Palade” University of Medicine, Pharmacy, Science and Technology of Târgu Mureș, 540139 Târgu Mureș, Romania; tiberiu.nyulas@umfst.ro; 5 Department of Cardiology, “George Emil Palade” University of Medicine, Pharmacy, Science and Technology of Târgu Mureș, 540139 Târgu Mureș, Romania

**Keywords:** left ventricular ejection fraction, nutritional status, acute myocardial infarction, CONUT score

## Abstract

There is currently little research on the effects of reduced left ventricular ejection fraction and altered nutritional status in patients with acute myocardial infarction. We therefore examined the interrelationship between the parameters of left ventricular dysfunction after acute myocardial infarction and changes in the Geriatric Nutrition Risk Index (GNRI) and the Nutrition Status Control Index (CONUT). Based on the evidence, frailty is considered to be an important factor affecting the prognosis of cardiovascular disease, so it is important to detect malnutrition early to prevent adverse cardiovascular events. This study was an observational, prospective study that included a total of 73 subjects who presented at the 3-month AMI follow-up. All subjects were subjected to laboratory tests and the groups were divided as follows: group 1, in which we calculated the CONUT score, (CONUT < 3 points, n = 57) patients with normal nutritional status and patients with moderate to severe nutritional deficiency (CONUT ≥ 3, n = 16). In group 2, the GNRI score was calculated and out of the 73 patients we had: GNRI ≥ 98, n = 50, patients with normal nutritional status, and GNRI < 98, n = 23, patients with altered nutritional status. The results of this study showed that we had significant differences between LVEF values at 3 months post-infarction where, in the CONUT group, patients with altered nutritional status had lower LVEF values (46.63 ± 3.27% versus 42.94 ± 2.54%, *p* < 0.001) compared to CONUT < 3. Also, in the GNRI group, we had lower LVEF values in patients with impaired nutritional status (46.48 ± 3.35% versus 44.39 ± 3.35%, *p* = 0.01). It can be seen that LVEF values are improved at 3 months post infarction in both groups, in patients with impaired nutritional status and in patients with good nutritional status. Patients with impaired nutritional status have lower ejection fraction and worse outcomes in both the CONUT and GNRI groups at 3 months post acute myocardial infarction.

## 1. Introduction

Hospitalized patients with several chronic diseases are frequently malnourished, which is linked to weakened immune systems, poor wound healing, and a worsening prognosis [1,2,3]. Research has consistently demonstrated that, despite this, clinical malnutrition has a negative impact on the healing process following illness, injury, or surgery. It is also typically linked to higher rates of morbidity and death in both acute and chronic conditions. Malnourished individuals stay in the hospital for far longer, and higher treatment expenses are associated with malnutrition [4]. It has been demonstrated that receiving proper nutritional care can lower hospitalization rates for malnutrition and associated expenses, therefore nutritional assessment is required to identify malnutrition early and start nutritional therapy on time [4].

The current system used to detect undernutrition in hospitals depends on the sensitivity of the doctor and not even 10% of cases requiring intervention are detected. A systematic and standardized approach is needed to identify this condition, and this is where nutritional screening tools play an important role. Screening tools such as the Nutrition Status Control Index (CONUT) and the Geriatric Nutritional Risk Index (GNRI) are effective for early detection and ongoing control of hospital undernutrition [5].

Apart from malnourishment, age and left ventricular (LV) systolic function continue to be significant predictors of survival following myocardial infarction in individuals [6]. In the general population with acute myocardial infarction, risk factors for adverse myocardial remodeling have included previous myocardial infarction, higher troponin levels, and LV ejection fraction at the time of myocardial infarction [7]; however, the need for more research is highlighted by the rising frequency of coronary artery disease risk factors and the particular difficulties associated with myocardial infarction in malnourished patients [8]. While it has been demonstrated that LV systolic function recovery following myocardial infarction predicts long-term outcomes, the prognostic significance of LV recovery in malnourished patients is not well understood [9]. Ejection fraction (EF) measured by echocardiography is considered the gold standard in the clinic; with all these limitations, echocardiography may have limited intra-observer and interobserver variability. According to Wu et al. [10], the coefficient of variation for LV structural function is up to 15% [11], restricting the slightest discernible meaningful change.

After myocardial infarction, left ventricular ejection fraction (LVEF) has both prognostic and therapeutic implications. This measurement is a performance benchmark recommended by both the American College of Cardiology and the American Heart Association.

The importance of this work lies in the fact that we evaluate the interrelationship between the alteration of ejection fraction at the time of myocardial infarction, 3 months after acute myocardial infarction (AMI) and nutritional status, taking into account the three control parameters of nutritional status, CONUT, and GNRI.

## 2. Materials and Methods

The present study includes 73 patients who presented for the 3-month AMI check-up at the Târgu Mureș Emergency County Clinical Hospital between 1 June and 15 August 2023. All 73 subjects had their CONUT and GNRI scores calculated to determine the degree of malnutrition and were divided as follows: In the CONUT group, we had patients with normal nutritional status or mild nutritional deficiency (CONUT < 3 points, n = 57), and patients with a dietary deficit that is mild to severe (CONUT ≥ 3, n = 16). In the GNRI group of the 73 patients, we had GNRI ≥ 98, n = 50, patients with normal nutritional status, and GNRI < 98, n = 23, patients with impaired nutritional status.

Demographic information and coronary risk factors were collected from patient observation charts. Blood samples were collected early in the morning after the overnight fast and dyslipidemia was defined as low-density lipoprotein (LDL-C) ≥ 140 mg/dL, high-density lipoprotein cholesterol (HDL-C) ≤ 40 mg/dL and triglycerides (TG) ≥ 150 mg/dL [12,13]. Also, from the blood samples, total blood cell count, blood glucose, serum albumin, creatinine, urea, aspartate aminotransferase (GOT), alanine aminotransferase (GPT), creatine kinase, ionogram, and gamma-glutamyltransferase (GGT) were determined, all of which were determined 3 months after admission. The left ventricular dysfunction parameters determined in this study were N-terminal B-type natriuretic peptide NTproBNP (pg/mL) determined at 1 day and 5 days after AMI using the PATHFAST™ (Polymedco, Cortlandt, NY, USA) equipment and LVEF (%). LVEF was measured by echocardiography and was determined both at 1 day after AMI and at the 3-month evaluation.

Assessment of nutritional status according to CONUT score was performed as shown in Table 1. A CONUT score < 3 indicates normal nutritional status, while a CONUT score ≥ 3 indicates moderate risk of malnutrition [4,14].

The GNRI score was calculated according to the formula:

GNRI = [14.98 albumin (g/L)] + [41.7 (weight/WLo)],

where WLo is the ideal weight and was calculated from the Lorentz equations: for males H − 100 − [(H − 150)/4]; for women H − 100 − [(H − 150)/2.5] (H: height) [15]. From these GNRI values, 2 grades of nutrition-related risk were classified: high risk of malnutrition (GNRI < 98) and (GNRI ≥ 98) normal nutritional status.

### 2.1. Ethics

This study was approved by the ethics committee of the “George Emil Palade” University of Medicine, Pharmacy, Science and Technology of Târgu Mureș, and the Ethics Committee of the Târgu Mureș Emergency Clinical County Hospital. The 1975 Helsinki Declaration’s ethical guidelines were followed in the conduct of this investigation. Every patient has given their written, informed consent for this study to be published.

### 2.2. Statistical Analysis

Comparisons were performed using the Mann–Whitney test for continuous variables and the chi-square test or Fisher’s exact test for categorical data. Continuous variables were represented as means ± standard deviation, medians, and ranges. Logistic regression analysis was performed to investigate the interrelationship between nutritional status according to the 2 scores and ejection fraction alteration in the AMI patient. The data were analyzed using Graph Pad InStat 3.10 software (GraphPad Software, Inc., San Diego, CA, USA), with a threshold for statistical significance set at *p* < 0.05 [16].

## 3. Results

The clinical characteristics of the total study sample by sex and age are shown in Table 2 and the demographic characteristics of patients after calculation of CONUT and GNRI score at 3 months after AMI are shown in Table 3. Also in Table 3, it can be seen that there are no statistically significant differences in terms of age either in the CONUT group (60.25 ± 10.29 years versus 57.69 ± 11.50 years; *p* = 0.39) or in the GNRI group (60.34 ± 11.61 years versus 58.26 ± 7.75 years; *p* = 0.37), but there were statistically significant differences in weight (76.00 ± 14.51 kg versus 84.43 ± 13.65 kg; *p* = 0.02) and body mass index (28.19 ± 4.24 kg/m^2^ versus 30.49 ± 2.95 kg/m^2^; *p* = 0.01) in the GNRI group. In the CONUT group, there were no significant differences in height, weight, BMI, or ideal weight, all with *p*> 0.05.

The laboratory biochemical data and blood cell counts in the CONUT and GNRI groups at 3 months post AMI are shown in Table 4. Paradoxically, urea and creatinine have higher values in the nutritionally deficient group compared to those with normal nutritional status in the CONUT group (all *p* < 0.05). Serum albumin values do not differ statistically significantly in the CONUT group, instead we have statistically significant differences in the GNRI group where values are lower in patients with impaired nutritional status (4.12 ± 0.23 g/dL versus 3.56 ± 0.14 g/dL; *p* < 0.001).

Lipid profile differs significantly in both the CONUT and GNRI groups, total cholesterol and LDL cholesterol values are lower among patients with altered nutritional status, and HDL cholesterol values are higher compared to CONUT < 3 and GNRI ≥ 98 (*p* < 0.01). Also, patients with an impaired status had lower triglyceride values in the CONUT group, where *p* = 0.002. Fasting blood glucose differed statistically significantly only in the GNRI group, where values were higher in patients with impaired nutritional status *p* = 0.02.

We assessed the parameters of left ventricular dysfunction immediately after acute myocardial infarction, and the NTproBNP values at 1 day and 5 days after infarction differed, but not statistically significantly, in both the CONUT group and the GNRI group. Left ventricular ejection fraction values at 1 day after infarction are also different in both groups, but not statistically significantly differently. What differs significantly are the LVEF values at 3 months post infarction, where in the CONUT group patients with impaired nutritional status had lower LVEF values (46.63 ± 3.27% versus 42.94 ± 2.54%, *p* < 0.001) compared to CONUT < 3. Also, in the GNRI group, we had lower LVEF values in patients with impaired nutritional status (46.48 ± 3.35% versus 44.39 ± 3.35%, *p* = 0.01). It can be seen that LVEF values improved at 3 months post infarction in both groups in patients with impaired nutritional status and in patients with good nutritional status. All of these results are shown in Table 5. The univariate and multivariate linear regression analysis of worsening LVEF are listed in Table 6.

**Figure 1:**
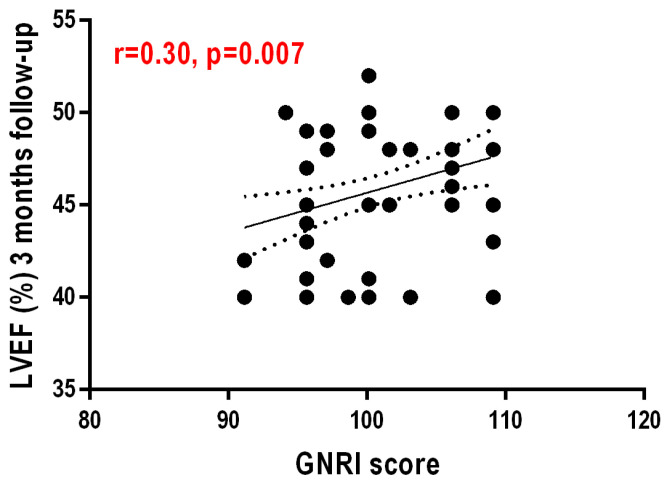
Correlation between nutritional status evidenced by GNRI score and left ventricular ejection fraction value at 3 months after AMI.

**Figure 2:**
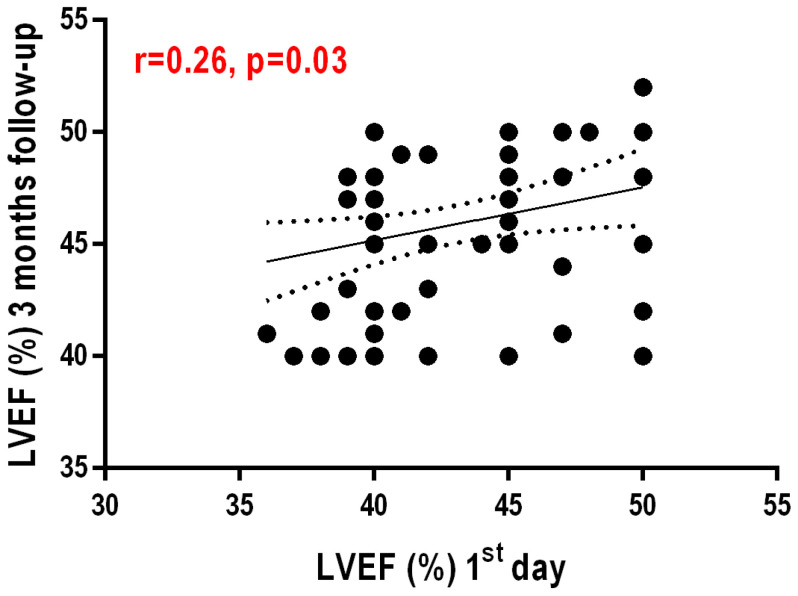
Correlation between left ventricular ejection fraction one day after AMI and ejection fraction 3 months after AMI.

The GNRI score values showed a moderately positive, significant correlation with LVEF at 3 months post infarction (r = 0.310, *p* = 0.008)—Figure 1. Another weakly positive but statistically significant correlation exists between LVEF at 1 day post infarction and LVEF at 3 months post AMI (r = 0.259, *p* = 0.027)—Figure 2.

**Figure 3:**
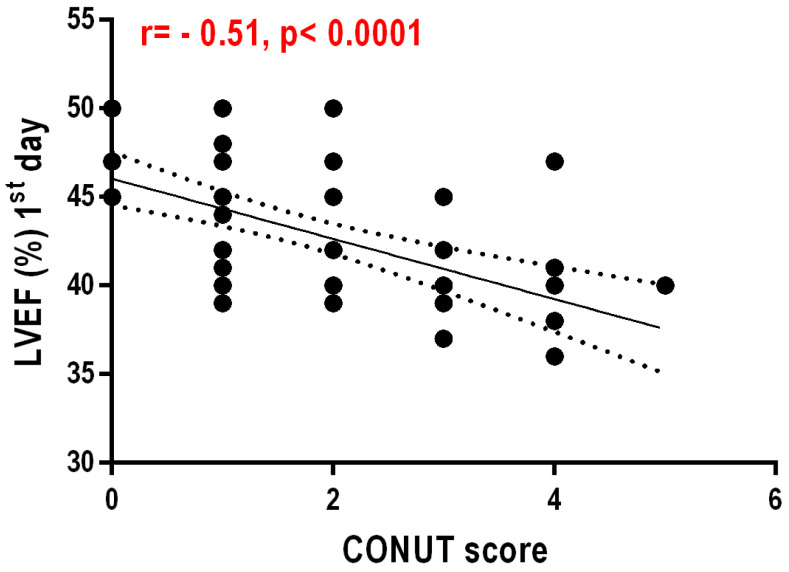
Correlation between left ventricular ejection fraction one day after AMI and CONUT score.

**Figure 4:**
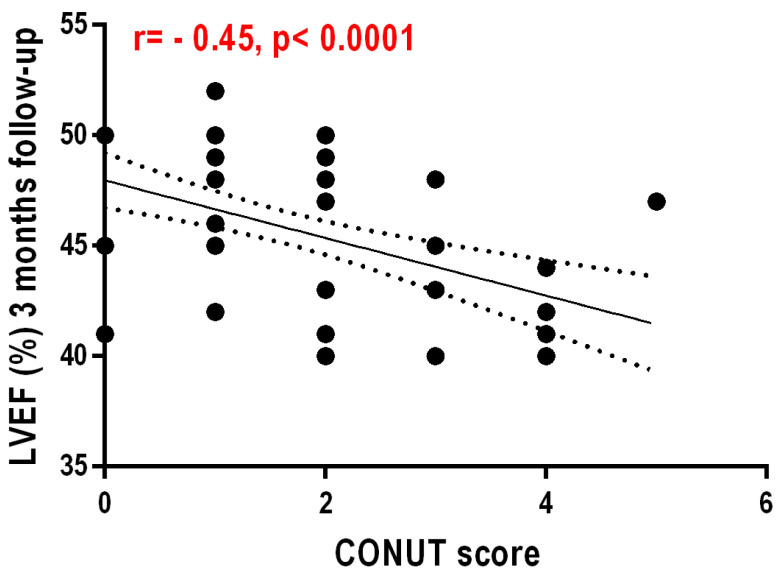
Correlation between left ventricular ejection fraction at 3 months after AMI and CONUT score.

Regarding the CONUT values and the correlation with the GNRI score values at 3 months after AMI, these are negative and statistically insignificant (r = −0.087, *p* = 0.464). Another weak but still statistically significant negative correlation exists between the CONUT score and LVEF at 1 day after AMI (r = −0.51, *p* < 0.0001)—Figure 3. CONUT values showed a weak but statistically significant negative correlation with LVEF at 3 months post infarction (r = −0.453, *p* < 0.0001)—Figure 4.

## 4. Discussion

Malnutrition is a frequent and significant problem and is seen particularly in elderly hospitalized patients, especially those with multiple chronic diseases. Even though it is quite difficult to determine the malnutrition status of the patient with AMI, it should be kept in mind that this problem may later lead to complications in terms of treatment and disease progression.

Left ventricular ejection fraction is an important determinant of the risk of heart failure and death in patients with myocardial infarction. The American College of Cardiology and the European Society of Cardiology recommend that LVEF be assessed in both ST-elevation (STEMI) and non-ST-elevation (NSTEMI) myocardial infarction patients before discharge [17,18,19,20,21].

This study aimed to investigate the correlation between left ventricular ejection fraction and nutritional status, expressed by CONUT and GNRI scores. A percentage of 21% showed malnutrition, calculated using the CONUT score, and 31% of them were identified as malnourished, calculated using the GNRI score. Patients with malnutrition in both CONUT and GNRI had lower LVEF values both at 1 day post AMI and 3 months post AMI.

The existence of a wide range of blood-based biomarkers to identify malnutrition status can help us to highlight clinical risk. According to recent studies, low serum albumin levels are common in patients with heart failure (25–33%) due to several factors including lifestyle, absorption disorders, a complex malnutrition–inflammation syndrome, which produces excessive oxidative stress [22,23,24,25,26].

And, in other studies, scores such as Prognostic Nutritional Index (PNI) and CONUT have been shown to be superior even in predicting mortality in patients with heart failure [27,28,29,30]. Another recently published study demonstrated the relationship between malnutrition and mortality in patients with LVEF ≥ 40%, as assessed by CONUT score in a cohort of patients undergoing coronary angiography [31]. Additionally, Iwakami et al. demonstrated that, within a group of 635 patients with acute heart failure, 78% of patients with an average age of 75 years in Japan had poor nutritional status based on the CONUT score [28]. Sze et al. demonstrated that worsening malnutrition was associated with worse outcomes in British heart failure patients using the three well-known scores, the GNRI, CONUT, and PNI scores [32]. Chen et al. showed that high CONUT values and neutrophil-to-lymphocyte ratio can be used to predict the occurrence of adverse clinical events and mortality in patients with AMI, and CONUT scores may be an independent predictor of major adverse cardiovascular events (MACEs) in patients with acute myocardial infarction [33]. An additional study on the relationship between nutritional status and recurrent major cardiovascular events after myocardial infarction showed that MACEs in post-MI patients were associated with nutritional status, which could be characterized by elevated inflammatory status and nutritional deficiency as determined by the CONUT score [34,35].

A study including 56 patients with AMI (44.64% with STEMI, 55.35% with NSTEMI) demonstrated that malnourished patients undergoing revascularization have an increased rate of in-hospital complications and a longer observation time in a tertiary cardiac intensive care unit, and assessment of nutritional status was calculated using the CONUT and PNI scores [36]. It has recently been shown that there is a strong relationship between low LVEF and the Naples Prognostic Score (NPS) in STEMI patients, with this study conducted on a cohort of 2.280 STEMI patients undergoing primary percutaneous coronary intervention between 2017 and 2022 [37]. Worsening nutritional status during hospitalization was linked to prognosis in patients with a low GNRI on admission, but not in patients with a high GNRI on admission, according to data from a recent study by Sunaga et al. that included 1.095 heart failure patients with preserved ejection fraction [38,39]. Our data indicate a correlation between parameters of left ventricular dysfunction after acute myocardial infarction and malnutrition status calculated using the CONUT and GNRI scores. LVEF had lower values in malnourished patients in both the CONUT and GNRI groups, and NTproBNP had lower values at both 1 day and 5 days after infarction in the CONUT ≥ 3 group.

The present study also has certain limitations, among which we first of all can list the small number of patients with acute myocardial infarction; however, while we were able to demonstrate that there is an association between altered nutritional status and left ventricular dysfunction, the follow-up period being a short one, only 3 months after AMI. In future studies, we may extend the follow-up period to observe differences in LVEF on day 1 post infarction in malnourished patients and 1 year post MI compared to patients with normal nutritional status. Secondly, we used only two screening scores for malnutrition, other scores we could have used include PNI or the Nutritional Risk Index (NRI). Third, patients included in the study were recruited from a single heart center, but we made every effort to enroll all patients with available data within the specified time frame. Further research including other biomarkers reflecting immunological activation such as troponin, tumor necrosis factor-α (TNF-α), interleukin (IL)-1, and (IL)-6, which may contribute to cardiac remodeling and left ventricular dysfunction by inducing cardiomyocyte hypertrophy, dilatation, fibrosis and apoptosis, may be of interest.

## 5. Conclusions

The correlation between the parameters of left ventricular dysfunction and nutritional status calculated by the two scores shows that patients with impaired nutritional status have worse outcomes at the 3-month post-infarction assessment, and lower values for LVEF (%) both at 1 day post infarction and at 3 months in patients with impaired nutritional status. Malnutrition is one of the risk factors for cardiovascular disease and in our study it was independently associated with adverse outcomes at 3 months post infarction. Future work would be needed to demonstrate the link between malnutrition and ventricular dysfunction.

## Figures and Tables

**Table 1:** Evaluation scores by CONUT [14].

Parameter	Score			
Serum albumin, g/dL	≥3.5	3.0–3.4	2.5–2.9	<2.5
Albumin score	0	2	4	6
Total cholesterol, mg/dL	≥180	140–179	100–139	<100
Cholesterol score	0	1	2	3
Total lymphocytes, count/mL	≥1600	1200–1599	800–1199	<800
Lymphocyte score	0	1	2	3

**Table 2:** Clinical characteristics of the total study sample by sex and age.

	Total	Female Gender (n = 29)	Male Gender (n = 44)	*p* Value	≤65 Years (n = 54)	>65 Years (n = 19)	*p* Value
**Demographic data and coronary risk factors**							
Age (years)	60.58 ± 9.5	60.38 ± 9.65	59.23 ± 11.17	0.65	55.96 ± 5.44	73.68 ± 5.36	**0.00**
Gender, male, n (%)	-	-	-	-	33 (61.1%)	11 (57.9%)	0.06
Smoking status, yes, n (%)	41 (56.2%)	16 (44.8%)	25 (56.8%)	0.89	27 (50.0%)	14 (73.7%)	0.07
Diabetes mellitus, n (%)	24 (32.9%)	12 (41.4%)	12 (27.3%)	0.20	18 (33.3%)	6 (31.6%)	0.88
Hypertension, n (%)	57 (78.1%)	23 (79.2%)	34 (77.2%)	0.57	18 (33.3%)	39 (66.7%)	0.40
Dyslipidemia, n (%)	28 (38.4%)	13 (44.8%)	15 (34.9%)	0.39	23 (43.4%)	5 (26.3%)	0.19
Hypercholesterolaemia n (%)	35 (47.9%)	33 (62.3%)	7 (36.8%)	**0.05**	28 (52.8%)	7 (36.8%)	0.23
Hypertriglyceridemia, n (%)	40 (54.8%)	19 (65.5%)	21 (48.8%)	0.16	33 (62.3%)	7 (36.8%)	**0.05**
**Renal function parameters**							
Urea, (mg/dL)	44.86 ± 32.5	47.45 ± 34.4	43.16 ± 31.47	0.58	34.57 ± 21.72	74.11 ± 40.22	**0.00**
Creatinine, (mg/dL)	1.13 ± 0.48	1.17 ± 0.53	1.11 ± −0.44	0.61	1.01 ± 0.41	1.47 ± 0.51	**0.00**
Uric acid, (mg/dL)	5.47 ± 2.13	5.66 ± 2.28	5.35 ± 2.04	0.56	4.76 ± 1.62	7.61 ± 2.09	**0.00**
eGFR, mL/min	86.67 ± 36.34	81.09 ± 33.17	89.82 ± 38.06	0.37	95.86 ± 34.49	66.37 ± 32.55	**0.00**
**Nutritional status**							
CONUT score	1.64 ± 1.2	1.52 ± 1.27	1.73 ± 1.16	0.47	1.63 ± 1.23	1.68 ± 1.15	0.86
GNRI score	100.73 ± 4.97	99.79 ± 4.89	101.34 ± 4.98	0.19	100.24 ± 4.75	102.11 ± 5.45	0.16
**Procedural characteristics**							
TIMI flow, III, n (%)	46 (63%)	19 (65.5%)	27 (61.4%)	0.93	38 (70.4%)	8 (42.1%)	0.08
Time to PCI, hours	4.05 ± 1.31	3.94 ± 1.14	4.14 ± 1.45	0.63	4.33 ± 1.27	3.36 ± 1.20	**0.03**
**Medication**							
Statins, n (%)	67 (91.8%)	25 (86.2%)	42 (95.5%)	0.15	52 (96.3%)	15 (78.9%)	**0.01**
P2y12 inhibitors, n (%)	69 (94.5%)	28 (96.6%)	41 (93.2%)	0.53	52 (96.3%)	17 (89.5%)	0.26
ACEI inhibitors, n (%)	70 (95.9%)	29 (100%)	41 (93.2%)	0.15	51 (94.4%)	19 (100%)	0.29
Beta blockers, n (%)	47 (64.4%)	22 (75.9%)	25 (56.8%)	0.09	33 (61.1%)	14 (73.7%)	0.32
Anticoagulants, n (%)	14 (19.2%)	4 (13.8%)	10 (22.7%)	0.34	10 (18.5%)	4 (21.1%)	0.80

The *p*-values are bolded when they are less than or equal to the significance level cut-off of 0.05.

**Table 3:** Demographic data in patients with score CONUT and GNRI.

	CONUT < 3 (n = 57)	CONUT ≥ 3 (n = 16)	*p* Value	GNRI ≥ 98 (n = 50)	GNRI < 98 (n = 23)	*p* Value
**Demographic data**						
Age (years)	60.25 ± 10.29	57.69 ± 11.50	0.39	60.34 ± 11.61	58.26 ± 7.75	0.37
Weight (kg)	78.02 ± 15.35	80.94 ± 12.21	0.43	76.00 ± 14.51	84.43 ± 13.65	**0.02**
Height (cm)	163.49 ± 8.09	168.56 ± 8.74	0.33	163.94 ± 8.64	166.04 ± 7.99	0.31
BMI (kg/m^2^)	29.03 ± 4.05	28.52 ± 3.93	0.65	28.19 ± 4.24	30.49 ± 2.95	**0.01**
Ideal weight	59.40 ± 6.36	63.69 ± 6.89	0.22	59.89 ± 6.79	61.32 ± 6.47	0.39

The information is presented as mean ± SD, with *p*-values denoting the body mass index, GNRI, and between-group CONUT. The *p*-values are bolded when they are less than or equal to the significance level cut-off of 0.05.

**Table 4:** Biochemical profile and blood cell count are presented in patients with CONUT and GNRI at 3 months after AMI.

	CONUT < 3 (n = 57)	CONUT ≥ 3 (n = 16)	*p* Value	GNRI ≥ 98 (n = 50)	GNRI < 98 (n = 23)	*p* Value
**Biochemical laboratory data**						
Urea (mg/dL)	40.23 ± 30.16	61.38 ± 36.10	**0.02**	43.06 ± 34.26	48.78 ± 28.65	0.46
Creatinine (mg/dL)	1.06 ± 0.47	1.31 ± 0.35	**0.03**	1.14 ± 0.48	1.07 ± 0.42	0.58
Uric acid (mg/dL)	5.31 ± 2.23	5.69 ± 1.99	0.52	5.42 ± 2.28	5.33 ± 1.96	0.85
Sodium (mmol/L)	138.34 ± 2.15	136.17 ± 0.94	**<0.001**	138.46 ± 2.23	136.57 ± 1.21	**<0.001**
Potassium (mmol/L)	4.51 ± 0.25	4.58 ± 0.47	0.46	4.48 ± 0.28	4.64 ± 0.34	**0.04**
Albumin (g/dL)	3.95 ± 0.27	3.89 ± 0.51	0.48	4.12 ± 0.23	3.56 ± 0.14	**<0.001**
Total cholesterol (mg/dL)	153.56 ± 23.91	124.63 ± 39.33	**<0.001**	146.42 ± 32.11	148.96 ± 26.15	0.72
Triglyceride (mg/dL)	135.91 ± 121.55	66.31 ± 58.71	**0.002**	133.44 ± 129.30	92.87 ± 65.54	0.08
Fasting blood glucose (mg/dL)	113.88 ± 22.03	119.94 ± 34.79	0.40	110.58 ± 21.25	125.26 ± 30.35	**0.02**
LDL cholesterol (mg/dL)	66.27 ± 21.94	43.80 ± 14.68	**<0.001**	65.71 ± 23.73	51.85 ± 16.34	**0.005**
HDL cholesterol (mg/dL)	55.84 ± 13.18	70.00 ± 32.13	**0.01**	52.42 ± 13.17	73.13 ± 23.80	**<0.001**
GGT (mg/dL)	59.38 ± 52.81	41.50 ± 7.87	**0.01**	55.37 ± 55.18	55.48 ± 23.47	0.99
CK (U/L)	148.79 ± 86.53	122.00 ± 66.10	0.19	166.53 ± 82.52	92.35 ± 57.74	**<0.001**
**Blood cell count**						
WBC (×10^3^/mm^3^)	7.98 ± 1.90	8.01 ± 0.68	0.91	7.91 ± 1.83	8.15 ± 1.40	0.53
RBC (×10^6^/mm^3^)	4.82 ± 0.40	4.61 ± 0.83	0.15	4.85 ± 0.43	4.62 ± 0.60	0.08
LYM (×10^3^/mm^3^)	1.96 ± 0.43	1.62 ± 0.38	**0.005**	1.92 ± 0.42	1.82 ± 0.47	0.39
NEU (%)	5.32 ± 1.76	5.64 ± 0.42	0.21	5.16 ± 1.54	5.90 ± 1.56	0.06
PLT (×10^3^/mm^3^)	240.75 ± 58.92	272.88 ± 71.50	0.07	239.12 ± 53.91	266.65 ± 76.77	0.08
Hb (g/dL)	14.68 ± 1.35	13.74 ± 3.10	0.08	14.87 ± 1.45	13.59 ± 2.41	**0.006**
Hct (%)	42.58 ± 4.16	40.68 ± 8.19	0.20	43.87 ± 3.71	38.45 ± 6.33	**<0.001**

Data are presented as means ± SD. GGT—gamma-glutamyl transferase; CK—creatine kinase; WBC—white blood cell; RBC—red blood cell; LYM—lymphocyte; NEU—neutrophile; PLT—platelet; Hb—hemoglobin; Hct—hematocrit. The *p*-values are bolded when they are less than or equal to the significance level cut-off of 0.05.

**Table 5:** Left ventricular dysfunction parameters in patients with CONUT and GNRI.

	CONUT < 3 (n = 57)	CONUT ≥ 3 (n = 16)	*p* Value	GNRI ≥ 98 (n = 50)	GNRI < 98 (n = 23)	*p* Value
**Left ventricular dysfunction parameters immediately following an acute myocardial infarction**						
NTproBNP (pg/mL) 1st day	2207.72 ± 5285.52	779.94 ± 1083.27	0.06	1549.19 ± 4391.57	2636.05 ± 5379.84	0.37
NTproBNP (pg/mL) 5th days	988.91 ± 1507.21	568.73 ± 548.39	0.13	895.49 ± 1362.85	933.43 ± 1452.49	0.92
LVEF (%) 1st day	39.35 ± 14.06	37.63 ± 10.40	0.65	41.04 ± 11.15	34.48 ± 16.46	0.05
**Left ventricular dysfunction parameter at 3 months follow-up**						
LVEF (%) 3 months follow-up	46.63 ± 3.27	42.94 ± 2.54	**<0.001**	46.48 ± 3.35	44.39 ± 3.35	**0.01**

The *p*-values are bolded when they are less than or equal to the significance level cut-off of 0.05.

**Table 6:** The univariate and multivariate linear regression analysis of worsening LVEF.

	Univariate Analysis		Multivariate Analysis	
Risk Factors	Coefficients	*p* Value	β-Coefficients	*p* Value
Age, years	−0.004	0.73	−0.07	0.58
CONUT score	−0.45	**0.0001**	−0.38	**0.005**
GNRI score	0.30	**0.03**	0.21	0.10
NTproBNP (pg/mL) 1st day	0.16	0.16	0.14	0.32
NTproBNP (pg/mL) 5th days	0.17	0.18	0.02	0.87

The *p*-values are bolded when they are less than or equal to the significance level cut-off of 0.05.

## Data Availability

Data are contained within the article.
